# Interaction of HSP20 with a viral RdRp changes its sub-cellular localization and distribution pattern in plants

**DOI:** 10.1038/srep14016

**Published:** 2015-09-11

**Authors:** Jing Li, Cong-Ying Xiang, Jian Yang, Jian-Ping Chen, Heng-Mu Zhang

**Affiliations:** 1 State Key Laboratory Breeding Base for Zhejiang Sustainable Pest and Disease Control, Key Laboratory of Biotechnology in Plant Protection of MOA and Zhejiang Province, Institute of Virology and Biotechnology, Zhejiang Academy of Agricultural Sciences, Hangzhou 310021, China; 2 College of Chemistry and Life Science, Zhejiang Normal University, Jinhua 321004, China

## Abstract

Small heat shock proteins (sHSPs) perform a fundamental role in protecting cells against a wide array of stresses but their biological function during viral infection remains unknown. Rice stripe virus (RSV) causes a severe disease of rice in Eastern Asia. OsHSP20 and its homologue (NbHSP20) were used as baits in yeast two-hybrid (YTH) assays to screen an RSV cDNA library and were found to interact with the viral RNA-dependent RNA polymerase (RdRp) of RSV. Interactions were confirmed by pull-down and BiFC assays. Further analysis showed that the N-terminus (residues 1–296) of the RdRp was crucial for the interaction between the HSP20s and viral RdRp and responsible for the alteration of the sub-cellular localization and distribution pattern of HSP20s in protoplasts of rice and epidermal cells of *Nicotiana benthamiana*. This is the first report that a plant virus or a viral protein alters the expression pattern or sub-cellular distribution of sHSPs.

Plant heat shock proteins (HSPs) are stimulated in response to a wide array of stress conditions and perform a fundamental role in protecting plants against abiotic stresses^1^,^2^,^3^. Generally, HSPs function as molecular chaperones, facilitating the native folding of proteins and preventing irreversible aggregation of denatured proteins during stress^4^,^5^. HSPs can be classified into five major categories based on molecular mass and sequence homology: HSPp100/ClpB, HSP90, 70 kDa heat shock protein (HSP70/DnaK), chaperonin (HSP60/GroEL), and small heat shock protein (sHSP). The sHSP family is one of the most abundant and complex groups and is characterized by a conserved α-crystallin domain (ACD) of 80–100 amino acids in the C-terminal region^3^,^6^. Most sHSPs are highly expressed under heat stress and often confer increased thermal tolerance by protecting proteins from irreversible denaturation^7^,^8^. The alpha-crystallin domain contains several beta-strands organized into two beta-sheets responsible for dimer formation, the basic building block of most sHSPs while other parts of the protein control oligomerisation, which is essential for sHSP function^8^. Heat shock granules that appear in the cell cytoplasm under stress conditions are largely composed of sHSPs together with the partially unfolded RNA-binding proteins and associated mRNAs that they are protecting^9^. Some studies have demonstrated that sHSPs act as ATP-independent molecular chaperones by binding proteins that are unfolding or denaturing and thereby preventing their aggregation and facilitating subsequent substrate refolding by ATP-dependent chaperone systems^6^,^8^,^10^,^11^. Although found in all domains of life, sHSPs are much more diverse in plants than in other organisms. Thus *Arabidopsis thaliana* has 19 and rice (*Oryza sativa*) has 23 sHSPs compared with 10 in humans, 4 in *Drosophila melanogaster*, and 2 in bacteria^6^. Plant sHSPs also protect cells against other environmental stresses, such as heavy metals, drought, cold, and oxidative stress^7^. There have been few reports of plant sHSP involvement in response to biotic stresses but it has been reported that a sHSP was induced in tobacco as a defense response to bacterial infection^12^. Stress granules are induced and then sometimes dispersed in some animal cells in response to virus infection^13^ but there have been no reports that SHSPs are involved in response to virus infection. In contrast, there are several reports that plant HSP70 proteins play roles during infection by various viruses including geminiviruses^14^,^15^ and potyviruses^16^.

Rice stripe disease is one of most devastating viral diseases of rice in East Asia^17^,^18^. Infected plants often have chlorotic stripes or mottling and necrotic streaks in the newly expanded leaves and growth is stunted^17^,^19^,^20^. The causal agent, Rice stripe virus (RSV), is one of best-studied rice viruses and is the type member of the genus *Tenuivirus*^21^. RSV is transovarially transmitted by small brown planthopper (SBPH) (*Laodelphax striatellus*) in a persistent and circulative-propagative manner^22^,^23^. Although RSV only infects rice and other poaceous plants in nature^22^, it can be transmitted experimentally to the model dicot *Nicotiana benthamiana*^24^,^25^.

The RSV genome consists of four single-stranded RNA segments, designated RNA1 to RNA4 in the decreasing order of their molecular weight, which encode seven ORFs using a negative or ambisense coding strategy^26^. RNA1 (~9 kb) is the largest RNA segment and has a single ORF in the viral-complementary sense, encoding a 337-kDa protein that is an RNA-dependent RNA polymerase (RdRp)^27^,^28^. The other three segments (RNA2, 3.5 kb; RNA3, 2.5 kb; RNA4, 2.2 kb) each have two open reading frames (ORFs) one on the viral RNA (vRNA) and the other on the viral complementary strand (vcRNA)^29^,^30^,^31^. RSV vRNA2 encodes a membrane-associated protein p2 that reportedly acts as an RNA silencing suppressor^32^. The vcRNA2 encodes a glycoprotein pc2 of unknown function but which moves from the ER to the Golgi bodies in a manner strictly dependent on COP I and COP II early secretion pathways^33^,^34^. RNA3 encodes an RNA silencing suppressor p3 from the vRNA and a nucleocapsid protein pc3 from the vcRNA^29^,^35^. RSV vRNA4 encodes a disease-specific protein p4 that accumulates in both infected plant and insect cells^36^. The protein encoded by vcRNA4 (pc4) has been identified as a cell-to-cell movement protein^24^. Infection by RSV selectively modifies the expression of host genes and establishes a complex interaction with host cell components to block cellular defense mechanisms and hijack host cell machinery^20^,^32^,^37^,^38^. RSV p2 can bind to OsSGS3, a rice host protein^32^, PsbP, a 23 kDa oxygen-evolving complex protein of plants, has been shown to interact with p4, and its silencing resulted in more severe symptoms and the accumulation of viral RNAs^37^. Recently, host HSP70 has been shown to interact with RSV RdRp, and RSV RNAs were reduced in HSP70-silenced *Nicotiana benthamiana*^38^.

In this study we investigated the interactions between sHSPs and RSV-encoded proteins and found that the expression and sub-cellular localization of a host small heat shock protein 20 (HSP20) was significantly affected by RSV infection and that this was caused by its interaction with the viral RdRp.

## Results

### Expression of a conserved small heat shock protein (sHSP) was regulated by viral infection

**Figure 1:**
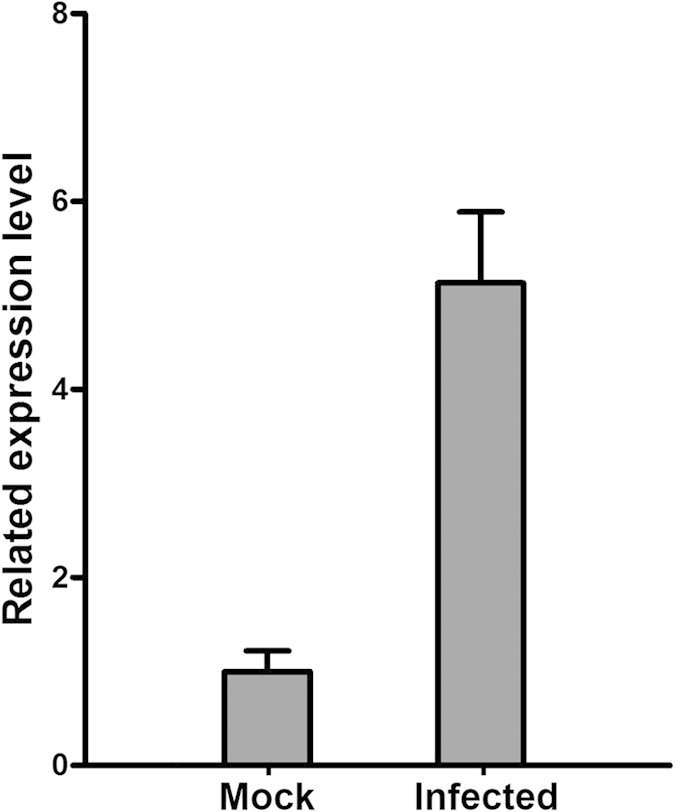
Quantitative RT-PCR analysis showing that transcripts of OsHSP20 were more abundant in RSV-infected rice plants.

Searches in both databases of rice expression profiles (http://cdna01.dna.affrc.go.jp and http://rice.plantbiology.msu.edu) showed that the expression of a rice small heat shock protein 20 (OsHSP20), encoded by Os03G026700, was significantly changed by infection with at least seven rice viruses, including RSV and also rice black-streaked dwarf virus (RBSDV), rice dwarf phytoreovirus (RDV), rice galled dwarf phytoreovirus (RGDV), rice grassy stunt tenuivirus (RGSV), rice tungro bacilliform virus (RTBV), and rice transitory yellowing virus (RTYV) (See Supplementary Materials Figure S1). Two published data sets with both Agilent and Affymetrix rice genome microarrays consistently showed that OsHSP20 accumulated in rice plants after RSV infection^20^,^39^, suggesting that OsHSP20 might be involved in the host response to RSV infection. We then performed quantitative RT-PCR (qPCR) and confirmed that OsHSP20 was significantly up-regulated by RSV infection in our rice plants (Fig. 1). *N. benthamiana* is an excellent experimental host of RSV that provides a simple viral inoculation system for further study of RSV-plant interactions^24^,^25^ and we confirmed that its HSP20 homologue was also up-regulated by RSV infection in *N. benthamiana* plants (data not shown). We then cloned both full-length coding regions of the OsHSP20 gene from rice and its homolog (NbHSP20) from *N. benthamiana* by RT-PCR with the respective primer pairs Os20F/Os20R and Nb20F/Nb20R (Table 1). Members of the HSP20 subfamily have 65–86% identity to one another and contain a conserved motif in the N-terminal part in addition to the characteristic sHSP alpha-crystallin domain (ACD) at the C-terminal region (See Supplementary Materials Figure S2).

### OsHSP20 and NbHSP20 self-interacted in yeast cells

**Figure 2:**
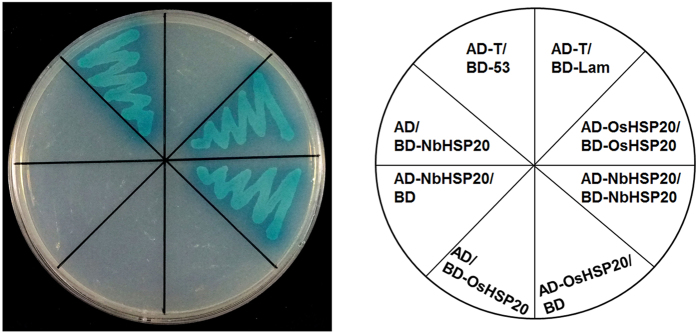
Self-interaction of OsHSP20 or NbHSP20 in yeast cells. Yeast colonies co-expressing BD-OsHSP20 and AD-OsHSP20, or BD-NbHSP20 and AD-NbHSP20 grew well and turned blue on SD/-Ade/-His/-Leu/-Trp/X-α-Gal/AbA medium as did yeast colonies expressing BD-53 with AD-T, which was used as the positive control. Yeast co-transformed with BD-Lam and AD-T, BD-OsHSP20 and AD, BD and AD-OsHSP20, BD-NbHSP20 and AD, or BD and AD-NbHSP20 were used as negative controls.

The full-length coding regions of OsHSP20 and NbHSP20 were separately cloned into the GAL4 binding domain vector pGBKT7 and activation domain vector pGADT7 and then combinations of plasmids expressing bait proteins BD-OsHSP20/AD-OsHSP20 or BD-NbHSP20/AD-NbHSP20 were co-transformed into *S. cerevisiae* Y2HGold cells after eliminating the autoactivation and toxicity. The resultant transformants were selected on SD/-Ade/-His/-Leu/-Trp/X-α-Gal/AbA medium. Both transformants of BD-OsHSP20/AD-OsHSP20 and BD-NbHSP20/AD-NbHSP20 as well as the positive control grew well and turned blue. In contrast, no growth was observed in the negative controls (Fig. 2). These results demonstrated that these HSP20 subfamily proteins self-interacted in yeast cells.

### OsHSP20 and NbHSP20 formed granules *in vivo* in the cytoplasm when expressed alone

**Figure 3:**
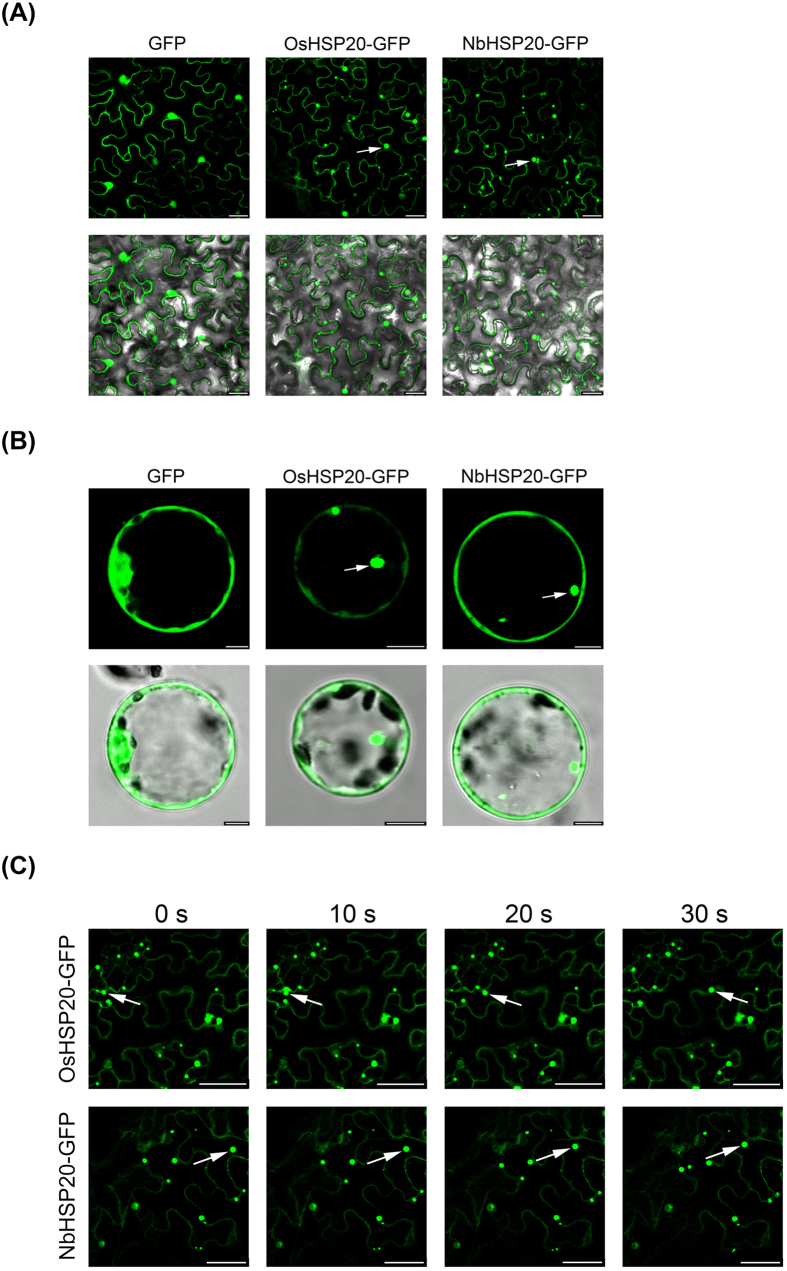
Sub-cellular localization of OsHSP20 and NbHSP20 proteins. (**A**) GFP fluorescence in *N. benthamiana* leaf epidermal cells agroinfiltrated with pCV-GFP-N1, pCV-OsHSP20-GFP and pCV-NbHSP20-GFP, respectively. The results were observed 48 h after infiltration. Scale bar, 25 μm. (**B**) GFP fluorescence in rice protoplasts transfected with pCV-GFP-N1, pCV-OsHSP20-GFP and pCV-NbHSP20-GFP, respectively. The results were observed 18 h after transfection. Scale bar, 5 μm. The white arrow points to a granule. The fluorescence and merged images are depicted in the upper and lower panels, respectively. (**C**) Images recording the movement of OsHSP20-GFP or NbHSP20-GFP in *N*. *benthamiana* epidermal cells. In each local field (upper and lower), four sequential pictures detecting green fluorescence were taken at 0, 10, 20 and 30 s. The mobile GFP granules are marked with white arrows. Scale bar, 50 μm.

To examine the sub-cellular localization of both OsHSP20 and NbHSP20 proteins, constructs expressing OsHSP20 or NbHSP20 fused with eGFP at their C terminus (pCV-OsHSP20-GFP and pCV-NbHSP20-GFP) were constructed and introduced into *N. benthamiana* epidermal cells by *Agrobacterium* infiltration. In confocal microscopy 2 days post-infiltration (dpi) GFP fluorescence was localized to numerous granules of various sizes in the cytoplasm of cells expressing OsHSP20-GFP or NbHSP20-GFP. No fluorescence was seen in the nucleus. In the control, the non-fused GFP was distributed generally in the cytoplasm and nucleus, which indicated that the moiety GFP did not affect the localization of OsHSP20-GFP or NbHSP20-GFP (Fig. 3A). The same results were obtained when the plasmids were delivered into rice protoplasts via polyethylene glycol (PEG) transfection (Fig. 3B).

In confocal microscopy, the OsHSP20-GFP and NbHSP20-GFP granules moved in the cytoplasm. To record the movement of OsHSP20-GFP or NbHSP20-GFP granules, four sequential photographs were taken over a period of 30 s (Fig. 3C). In addition, a video recording the movement of OsHSP20-GFP and NbHSP20-GFP under the GFP channel was taken. The granules moved at different speeds and often moved into another focal plane (See Supplementary Materials Videos S1 and S2).

### Distribution patterns of both OsHSP20 and NbHSP20 were affected by RSV infection

**Figure 4:**
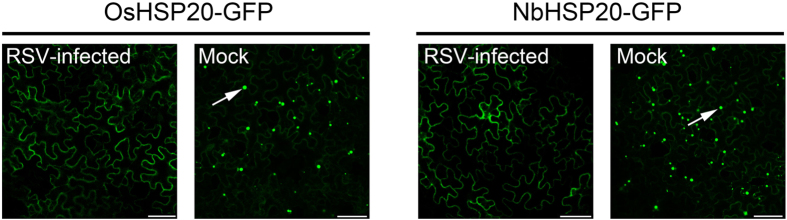
Localization of OsHSP20 or NbHSP20 was affected by RSV infection. GFP fluorescence in healthy (Mock) or RSV-infected *N. benthamiana* leaf epidermal cells agroinfiltrated with pCV-OsHSP20-GFP (left) and pCV-NbHSP20-GFP (right), respectively. The white arrow points to a granule. The results were observed 48 h after infiltration. Scale bar, 50 μm.

To further determine the relationship between the HSP20s and RSV infection, plasmids expressing OsHSP20-GFP or NbHSP20-GFP were introduced into healthy or RSV-infected *N. benthamiana* epidermal cells by *Agrobacterium* infiltration. Both proteins were localized in the cytoplasm of RSV-infected cells (Fig. 4) and no fluorescence was seen in the nucleus, as in healthy cells (Fig. 3). However, while numerous granules with a variety of sizes were observed in the cytoplasm of non-infected cells almost no GFP granules were detectable in the cytoplasm of RSV-infected cells (Fig. 4).

### Both HSP20s interacted with RSV RdRp

**Figure 5:**
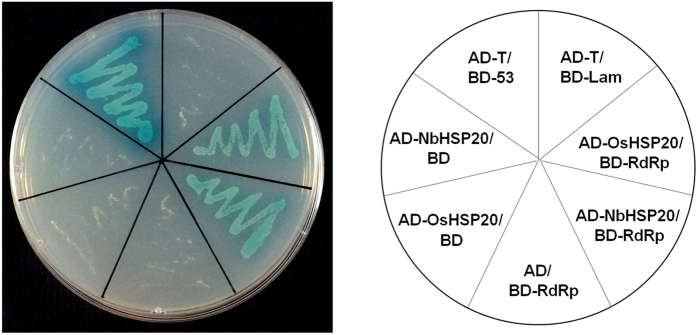
Interactions of RSV RdRp with the OsHSP20 or NbHSP20 in co-transformed yeast cells grown on SD/-Ade/-His/-Leu/-Trp/X-α-Gal/AbA. Yeast colonies expressing BD-RdRp with AD-OsHSP20 or AD-NbHSP20 were able to grow well and turned blue on SD/-Ade/-His/-Leu/-Trp/X-α-Gal/AbA medium as did yeast colonies expressing BD-53 with AD-T, which was used as the positive control. Yeast co-transformed with BD-Lam and AD-T, BD-RdRp and AD, BD and AD-OsHSP20, or BD and AD-NbHSP20 were used as negative controls.

To investigate the interaction between the HSP20 and viral proteins, the full-length coding regions of OsHSP20 and NbHSP20 were cloned into the GAL4 binding domain vector pGBKT7 as bait for screening a prey library of RSV cDNA in yeast two-hybrid (YTH) assays. More than 10 independent clones were recovered following growth on selective media and sequences of all these clones were 100% identical to RSV genomic segment RNA1 encoding the viral RdRp. Combinations of plasmids expressing bait proteins BD-OsHSP20 or -NbHSP20 and prey protein AD-RdRp were then co-transformed into *S. cerevisiae* using the plasmid combinations BD-Lam/AD-T, BD/AD-RdRp, BD/AD -OsHSP20 or BD/AD -NbHSP20 as negative controls and BD-53/AD-T as a positive control. Only transformants of BD-OsHSP20/AD-RdRp, BD-NbHSP20/AD-RdRp and the positive control grew well on the selective medium and turned blue. In contrast, no growth was observed in the negative controls (Fig. 5). These results consistently indicated a strong interaction between the HSP20 proteins and RSV RdRp.

### The N-terminal part of RSV RdRp was responsible for its interactions with OsHSP20 and NbHSP20 *in vivo* and *in vitro*

**Figure 6:**
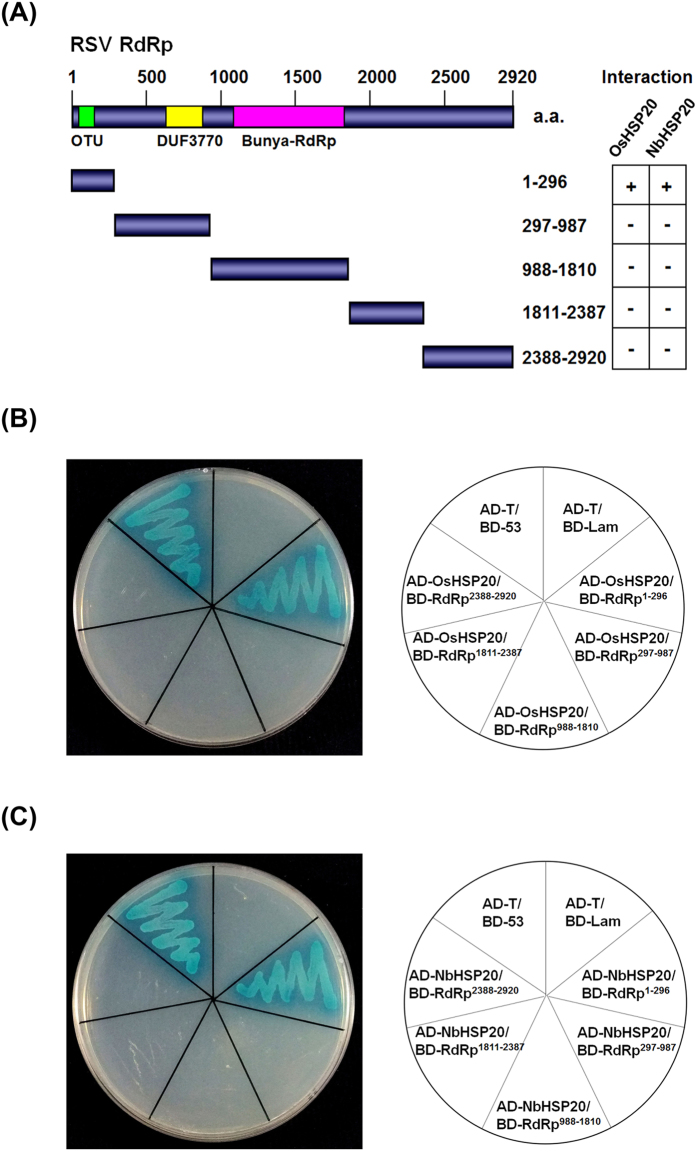
Interactions of OsHSP20 or NbHSP20 with fragments of RSV RdRp in co-transformed yeast cells grown on SD/-Ade/-His/-Leu/-Trp/X-α-Gal/AbA. (**A**) Five peptides were selected to cover the whole RSV RdRp based around the conserved domains (OUT, DUF3770 and Bunya-RdRp domain) of the protein. The numbers denote RSV RdRp amino acid positions. The ability of RSV RdRp fragments to interact with both OsHSP20 and NbHSP20 in YTH assays is shown on the right (+, positive; −, negative). (**B**) Yeast colonies expressing BD-RdRp^1–296^ with AD-OsHSP20 grew well on the selective medium, but those expressing BD-RdRp^297–987^, BD-RdRp^988–1810^, BD-RdRp^1811–2387^ or BD-RdRp^2388–2920^ with AD-OsHSP20 did not. (**C**) Yeast colonies expressing BD-RdRp^1–296^ with AD-NbHSP20 grew well on the selective medium, but those expressing BD-RdRp^297–987^, BD-RdRp^988–1810^, BD-RdRp^1811–2387^ or BD-RdRp^2388–2920^ with AD-NbHSP20 did not. Yeast co-transformed with BD-53 and AD-T, and BD-Lam and AD-T were used as the positive and negatives control, respectively.

The large RdRp protein of RSV has multiple functional motifs and domains^40^. Five fragments of the RdRp, divided on the basis of the conserved domains (RdRp^1–296^, RdRp^297–987^, RdRp^988–1810^, RdRp^1811–2387^ and RdRp^2388–2920^), were then tested separately by cloning into the yeast vectors to determine which part was involved in the interaction with HSP20 (Fig. 6A). Only transformants expressing BD-RdRp^1–296^/AD-OsHSP20, BD-RdRp^1–296^/AD-NbHSP20 and the positive control grew well on the selective medium and turned blue, suggesting that the N-terminal part of RdRp should be crucial for the interaction between the HSP20s and the viral RdRp (Fig. 6B,C).

**Figure 7:**
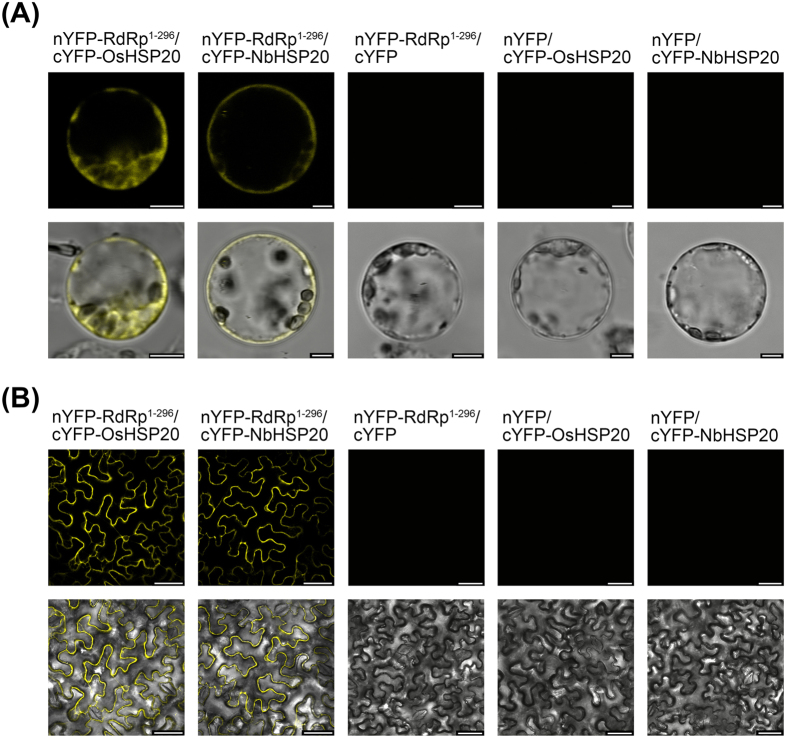
Interactions between the N-terminus (residues 1-296) of RSV RdRp and host HSP20 in living plant cells. (**A**) Visualization of RdRp^1–296^-HSP20 interaction in rice protoplasts by BiFC assay. Rice protoplasts were co-transfected with recombinant BiFC vectors containing the constructs indicated above the images. The results were observed 18 h after transfection. Scale bar, 5 μm. (**B**) Visualization of RdRp^1–296^-HSP20 interaction in *N*. *benthamiana* epidermal cells by BiFC assay. *N*. *benthamiana* leaves were co-infiltrated with recombinant BiFC vectors containing the constructs indicated above the images. The results were observed 48 h after infiltration. Scale bar, 50 μm. The fluorescent and merged images are depicted in the upper and lower panels, respectively.

To verify the YTH results, we used bimolecular fluorescence complementation (BiFC) assays to test the *in vivo* interactions between the N-terminus (residues 1–296) of RSV RdRp and host HSP20 in living plant cells. The coding sequence of RdRp^1–296^ was cloned into the vector pCV-nYFP-C and that of OsHSP20 or NbHSP20 into pCV-cYFP-C to generate plasmids pCV-nYFP-RdRp^1–296^, pCV-cYFP-OsHSP20 and pCV-cYFP-NbHSP20, respectively. Rice protoplasts were transfected with pCV-nYFP-RdRp^1–296^/pCV-cYFP-OsHSP20 and pCV-nYFP-RdRp^1–296^/pCV-cYFP-NbHSP20, while the pairs pCV-nYFP-RdRp^1–296^/pCV-cYFP, pCV-nYFP/pCV-cYFP-OsHSP20 and pCV-nYFP/pCV-cYFP-OsHSP20 were used as the negative controls. *N. benthamiana* leaves were transformed by co-infiltration with *A. tumefaciens* C58C1 cells harbouring the same combinations. Samples were examined for YFP fluorescence using confocal laser scanning microscopy. As shown in Fig. 7, co-expression of pCV-nYFP-RdRp^1–296^/pCV-cYFP-OsHSP20 or pCV-nYFP-RdRp^1–296^/pCV-cYFP-NbHSP20 in both rice protoplasts (18 h after transfection) (Fig. 7A) and *N. benthamiana* leaf cells (48 h after infiltration) (Fig. 7B) resulted in a strong YFP fluorescence in the cytoplasm and nucleus, whereas no fluorescence was observed in the negative controls.

**Figure 8:**
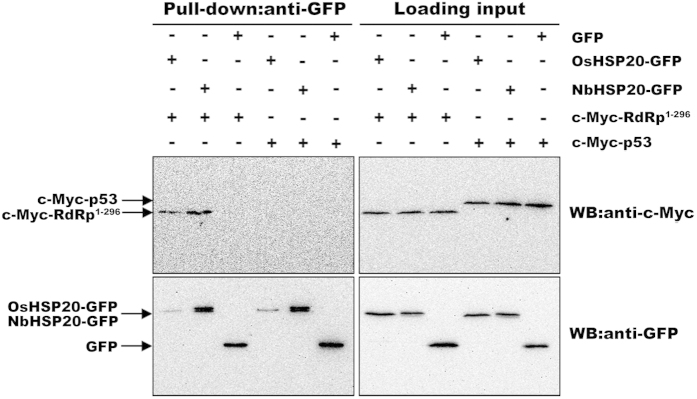
*In vitro* pull-down analysis of interactions between the N-terminus of RSV RdRp and host HSP20. *In vitro* translated c-Myc-RdRp^1–296^ or c-Myc-p53 fusion protein was incubated with equal amount of OsHSP20-GFP, NbHSP20-GFP or GFP, and accreted to GFP-Trap M beads. Beads were washed and analyzed by western blot assays using an anti-c-Myc antibody (upper) or an anti-GFP antibody (lower). The two right membranes show the inputs of *in vitro* translated proteins in pull-down assays. Black arrows indicate the corresponding bands of c-Myc-p53, c-Myc-RdRp^1–296^, OsHSP20-GFP, NbHSP20-GFP and GFP, respectively.

Interactions between the N-terminus of RSV RdRp and host HSP20 were further confirmed through *in vitro* pull-down assays. Equal amounts of *in vitro* translated c-Myc-RdRp^1–296^ and OsHSP20-GFP or NbHSP20-GFP were mixed and then pulled down with the GFP-Trap M beads followed by western blot assays using an anti-c-Myc or anti-GFP antibody. In these experiments, combinations of OsHSP20-GFP and c-Myc-p53, NbHSP20-GFP and c-Myc-p53, GFP and c-Myc-RdRp^1–296^, and GFP and c-Myc-p53 were used as controls. Immunoblot analyses using an anti-c-Myc antibody demonstrated that c-myc-RdRp^1–296^ was pulled down by OsHSP20-GFP and NbHSP20-GFP, but not by the control GFP (Fig. 8, top), although the anti-GFP antibody did pull-down OsHSP20-GFP, NbHSP20-GFP and control GFP proteins (Fig. 8, bottom). These experiments clearly demonstrated that RdRp^1–296^ bound to both OsHSP20-GFP and NbHSP20-GFP *in vitro*. Thus these results consistently indicated that the N-terminal part of RSV RdRp was responsible for its interactions with OsHSP20 or NbHSP20 *in vivo* and *in vitro*.

### Both sub-cellular localization and distribution patterns of OsHSP20 and NbHSP20 were affected by the N-terminal part of RSV RdRp

**Figure 9:**
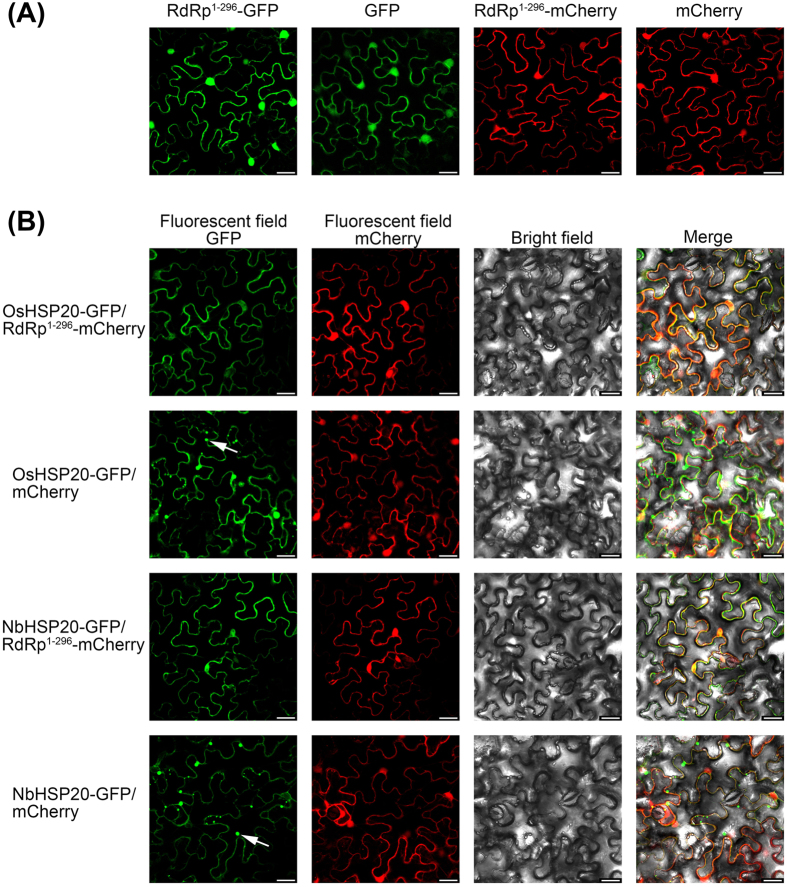
Localization of OsHSP20 or NbHSP20 was affected by expression of the N-terminus of RSV RdRp. (**A**) Sub-cellular localization of RSV RdRp^1–296^ fused with GFP or mCherry in *N. benthamiana* leaf epidermal cells. (**B**) Epidermal cells of *N. benthamiana* transiently co-expressing RdRp^1–296^-mCherry and OsHSP20-GFP or NbHSP20-GFP. The white arrow points to a granule. The results were observed 48 h after infiltration. Scale bar, 25 μm.

To determine whether the sub-cellular alteration of HSP20s was associated with that of RSV RdRp, plasmids expressing RdRp^1–296^-GFP and RdRp^1–296^-mCherry were first constructed and introduced into *N. benthamiana* epidermal cells by *Agrobacterium* infiltration. Fluorescence microscopy indicated that both RdRp^1–296^-GFP and RdRp^1–296^-mCherry resulted in a pattern of diffuse and uniform fluorescence in the cytoplasm and nucleus at 2 dpi (Fig. 9A). When RdRp^1–296^-mCherry was co-expressed with either OsHSP20-GFP or NbHSP20-GFP, the two proteins co-localized in a pattern identical to that formed by RdRp^1–296^-mCherry alone (Fig. 9B) and no GFP granules were seen in the cytoplasm. Control combinations ruled out the possibility that GFP or mCherry expression might have some aberrant effects on the distribution of RdRp1^–296^-mCherry, OsHSP20-GFP or NbHSP20-GFP. The co-localization of RdRp^1–296^ and HSP20 confirmed that the interaction between RSV RdRp and HSP20 mediated a dramatic effect on the distribution of HSP20. The sub-cellular distribution of GFP-fused OsHSP20 or GFP-fused NbHSP20 when co-expressed with RSV RdRp was very similar to that in RSV-infected cells, suggesting that RSV RdRp was responsible for both sub-cellular localization and distribution patterns of OsHSP20 and NbHSP20 in infected plants.

## Discussion

sHSPs, defined by possessing a conserved α-crystallin domain (ACD), are the most abundant and complex subset of HSPs in plants^41^. A key function of the sHSPs is to prevent aggregation of denatured proteins. By forming a soluble complex with substrate proteins, they can create a transient reservoir of substrates for subsequent refolding by ATP-dependent chaperone systems^6^,^8^,^10^,^11^. Rice has a total of 14 sHSPs subfamilies, which are predicted to locate to various cellular organelles, including the cytosol, nucleus, chloroplasts, mitochondria, endoplasmic reticulum, and peroxisomes^42^. Organelle-targeted sHSPs are unique to plants and their diverse functions are thought to be associated with their sub-cellular localization^43^ although little is known about their distinct functions^41^. Here both OsHSP20 and NbHSP20 were shown to be localized within the cytosol (Fig. 3), which was consistent with the predicted localization of the Class I (CI) subfamily of sHSPs^42^, the subfamily of which both HSP20s appear to be members. Sequence analysis showed that such HSP20s from a variety of plant species contained a highly conserved motif in the N-terminal part that has not previously been described in addition to the characteristic ACD domain of sHSPs^43^. The novel conserved motif “DPFSLDVWDPF” may act as a signal involved in their localization. Within the cytosol, both GFP-fused OsHSP20 and NbHSP20 formed fluorescent granules of different sizes and shapes when expressed alone in *N. benthamiana* epidermal cells or rice protoplasts (Fig. 3) and strong self-interactions were always observed in yeast colonies co-expressing BD-OsHSP20/AD-OsHSP20, or BD-NbHSP20/AD-NbHSP20 (Fig. 2), suggesting that both HSP20 proteins could function *in vivo* as dimers or larger oligomers. This was consistent with many previous reports showing that some sHSPs formed large oligomers *in vitro* from multiple subunits^41^,^44^,^45^. Interestingly, the fluorescent granules formed by GFP-fused OsHSP20 and NbHSP20 were able to move within the cytosol, suggesting that they might interact with other components of the plants. Most likely, these cytoplasmic granules were aggregates consisting mainly of oligomerized sHSPs, which could be, to a degree, similar in structure and composition to those of heat shock granules formed by accumulation of HSPs under hyper-thermic condition^9^. Further work is needed to identify those components and help understand the significance of these granules.

Interactions between host plant proteins and viral components are presumed to play an important role in the RSV life cycle or in viral pathogenicity. In addition to interactions between RSV or specific RSV gene products and host proteins that have already been reported^32^,^37^,^38^, we have now shown that the expression pattern and sub-cellular distribution of an OsHSP20 gene was regulated by RSV infection. This is the first report that sHSPs could be manipulated by a plant virus. A series of *in vivo* and *in vitro* protein-protein interaction assays further showed that the HSP20 interacted with the RSV RdRp and is the first report of a sHSP-plant viral RdRp interaction.

In the YTH assays, host HSP20 proteins interacted specifically with the N-terminus (residues 1–296) of RSV RdRp (Fig. 6). This segment contains a viral ovarian tumour (OTU) domain near its N-terminus which is thought to have deubiquitination activity^40^,^46^. The deubiquitylating function of viral RdRp has been suggested to provide a way to interfere with the proteolytic pathway in host plants during viral infection^40^,^46^. This might be a novel strategy of viral pathogenicity considering that sHSPs function as important players in regulating cellular proteostasis^47^. A previous study reported that RSV pc4 interacted with a small heat shock protein (EU325986) from Wuyujing 3, a susceptible *japonica* rice cultivar^48^, in yeast cells. However, in our YTH assays, only interaction between the OsHSP20 and viral RdRp was detected. To determine whether the HSP20 interacted with RSV pc4, we also investigated the pc4-OsHSP20 or pc4-NbHSP20 interaction by YTH assays, but no interaction was detected between the two proteins (Figure S3). Nucleotide sequence analysis indicated that pc4 gene sequences varied among RSV isolates from different geographical origins^49^, which might explain why pc4 did not interact with OsHSP20 or NbHSP20 in our experiments.

In transient expression systems, GFP fluorescence appeared as granular structures in the cytoplasm when HSP20 was expressed alone in *N. benthamiana* epidermal cells or rice protoplasts (Fig. 3). However, no GFP granules or reconstituted YFP-fluorescent granules were detected when the N-terminus of RSV RdRp and HSP20 were co-expressed in the leaves of *N*. *benthamiana* or in rice protoplasts (Figs 7 and 9), and the RdRp^1–296^/HSP20 complex had a diffuse distribution pattern in the cytoplasm and nucleus (Fig. 7), similar to that in *N*. *benthamiana* epidermal cells co-expressing RdRp^1–296^-mCherry/OsHSP20-GFP or RdRp^1–296^-mCherry/NbHSP20-GFP (Fig. 9). These results consistently suggested that the strong interactions between the N-terminus of RSV RdRp and HSP20 significantly affected the sub-cellular localization and distribution pattern of host HSP20 proteins. We hypothesize that the hetero-interaction between the HSP20 and viral RdRp destroyed the self-interaction of HSP20, leading to the disappearance of fluorescent granules and the diffuse distribution pattern of HSP20. If sub-cellular localization and self-interaction *in vivo* is important for its chaperone activities, it would be expected that this distributional change would affect the function of HSP20. In addition, RSV infection also affected the sub-cellular distribution of host HSP20 proteins. The GFP granules were not present if OsHSP20-GFP or NbHSP20-GFP were expressed in the cytoplasm of RSV-infected *N. benthamiana* epidermal cells (Fig. 4).

Previous research has revealed that some plant HSPs (especially HSP70s) were involved in the viral life cycle, including cell entry, virion assembly, the transfer of the viral genome segments into the nucleus, replication of the viral genome, morphogenesis of the virion particles and transformation of the cell^4^,^50^. However, the biological function of plant sHSPs in viral infection has been unknown. We have now demonstrated that the host HSP20 proteins interacted with the viral RdRp of RSV and that the N-terminal part of the RdRp was crucial for this interaction. Further experimental work will be needed to understand the significance of this interaction but the fact that the viral RdRp interferes with the self-interaction of the host HSP20 suggested that the virus might suppress the formation of stress granules that could potentially be an anti-viral response. Stress granules are initially induced but then dispersed in animal cells infected by poliovirus^51^ and this could be a broadly similar effect. Alternatively (or additionally), it is interesting that a previous study showed that HSP70 is necessary for RSV infection and that HSP70 also interacts with the N-terminal part of RSV RdRp. This may suggest that interactions between various HSPs and the viral RdRp play an important role in viral replication. These findings are therefore a step forward in understanding a virus that causes a seriously damaging disease of one of the most important crop plants in the world.

## Materials and Methods

### Plant materials and growth conditions

*Nicotiana benthamiana* plants were grown in 10-cm pots filled with a mixture of 60% vermiculite and 40% meadow soil and mechanically inoculated at the six-leaf stage with crude extracts from RSV-infected *O. sativa* leaves, as described previously^52^. Rice (*O. sativa*) was grown by germinating seeds on mesh supported in plastic containers containing ½ strength Murashige and Skoog (MS) nutrient solution. All plants used in this study were grown in a growth chamber at 25 °C with 16 h light/8 h dark and 70% r.h.

### Cloning and sequencing of a rice sHSP and its homolog in *Nicotiana benthamiana*

The coding sequence of intact OsHSP20 was amplified by PCR with the primer pair Os20F/Os20R (Table 1) from a leaf cDNA library of rice cv. Nipponbare plants, ligated into the pGEM-T vector (Promega, Madison, WI, USA), and then transformed into competent *Escherichia coli* DH5α. The recombinant plasmid DNA (pGEM-OsHSP20), used for sequencing was prepared using the QIAprep spin mini prep kit (Qiagen), and the inserts were sequenced entirely on both strands using the BigDye Terminator v3.1 Cycle Sequencing Kit (Perkin Elmer Applied Biosystems, Foster, USA) on an ABI PRISM 3730 DNA Sequencer (Perkin Elmer Applied Biosystems, Foster, USA) with universal primers T7 and SP6. The sequence of HSP20 gene from *N. benthamiana* was searched on the website of the SOL genomic network (http://solgenomics.net). The full-length coding sequence of a homologue of OsHSP20 in *N. benthamiana* (Niben.v0.4.2.Scf54613, named as NbHSP20 in this study) was cloned with the primer pair Nb20F/Nb20R (Table 1) from *N. benthamiana* leaf cDNA and the resultant recombinant plasmid DNA (pGEM-NbHSP20) was sequenced as described above. Sequence assembly and analysis was performed using the DNAman version 6.0 program (Lynnon BioSoft, Quebec, Canada).

### Yeast two-hybrid (YTH) assays

The yeast GAL4 binding domain vector pGBKT7 and GAL4 activation domain vector pGADT7 (Clontech, Palo Alto, CA) were used for yeast two hybrid (YTH) assays. RSV- Zhejiang isolate and its cDNA library were from our laboratory^40^. To construct plasmids for YTH analysis, the coding sequence of OsHSP20 was digested from the recombinant plasmids pGEM-OsHSP20 with the restriction enzymes *Nde*I and *Bam*HI and then inserted into the *Nde*I/*Bam*HI sites of yeast GAL4 binding domain pGBKT7 and activation domain pGADT7 vectors, creating the recombinant bait plasmid BD-OsHSP20 and prey plasmid AD-OsHSP20, respectively. The coding sequence of NbHSP20 was digested from the recombinant plasmid pGEM-NbHSP20 with restriction enzymes *Nde*I and *EcoR*I, and then inserted into the *Nde*I/*EcoR*I sites of pGBKT7 and pGADT7 vectors, creating the recombinant plasmids BD-NbHSP20 (bait) and AD-NbHSP20 (prey).

In further YTH assays, the coding sequences of five fragments of RSV RdRp (1–296, 297–987, 988–1810, 1811–2387 and 2388–2920) were amplified separately from RSV cDNA using primer pairs R1-1F/R1-1R, R1-2F/R1-2R, R1-3F/R1-3R, R1-4F/R1-4R and R1-5F/R1-5R (Table 1), respectively. The products were then inserted into the *Nde*I/*Bam*HI, *Nde*I/*Eco*RI or the unique *Bam*HI site of pGBKT7 and pGADT7, creating the recombinant bait and prey plasmids, BD- and AD-RdRp^1–296^, -RdRp^297–987^, -RdRp^988–1810^, -RdRp^1811–2387^ and -RdRp^2388–2920^, respectively.

Yeast transformation and library screening were conducted in accordance with the recommended procedures (Matchmaker Gold Yeast Two-Hybrid System; Yeastmaker Yeast Transformation System 2, Clontech). Briefly, the RSV cDNA library was screened using BD-OsHSP20 and -NbHSP20 as the baits in *Saccharomyces cerevisiae* strain Y2HGold (Clontech). Yeast transformants were selected on a synthetic defined medium lacking Ade, His, Leu, and Trp (SD/-Ade/-His/-Leu/-Trp) and transferred onto synthetic defined medium lacking Ade, His, Leu, and Trp with 40 μg ml^−1^ X-α-Gal and 70 ng ml^−1^ AbA (SD/-Ade/-His/-Leu/-Trp/X-α-Gal/AbA). The positive candidate plasmids containing the viral cDNAs were isolated and determined by sequencing. Protein-protein interactions were confirmed in yeast by co-transformation into *S. cerevisiae* strain Y2HGold with bait and prey plasmids^53^. Co-transformants were first plated on SD/-Ade/-His/-Leu/-Trp medium, and positive yeast colonies that grew on the auxotrophic medium were then tested for α-galactosidase activity on SD/-Ade/-His/-Leu/-Trp/X-α-Gal/AbA medium. BD-53 and AD-T were also co-transformed as a positive control, while BD-Lam and AD-T were co-transformed as a negative control. Three independent experiments were performed to confirm the results.

### *In vitro* pull-down assay and western blot analysis

For the *in vitro* pull-down assay, the fusion gene c-Myc-RdRp^1–296^ was amplified by PCR with primer pair P1-1F/P1-1R (Table 1) using BD-RdRp^1–296^ as template and was digested with *Kpn*I/*Not*I and cloned into the same sites of the pCMVTNT expression vector (Promega, Madison, WI, USA), producing pCMV:c-Myc-RdRp^1–296^. The fusion genes OsHSP20-GFP and NbHSP20-GFP were amplified by PCR with primer pairs POs20F/POs20R and PNb20F/PNb20R (Table 1) using pCV-OsHSP20-GFP and pCV-NbHSP20-GFP as templates, respectively. The amplified PCR products were digested with *Eco*RI/*Kpn*I or *Eco*RI/*Not*I, and cloned into the same sites of pCMVTNT, producing pCMV:OsHSP20-GFP and pCMV:NbHSP20-GFP, respectively. GFP and c-Myc-p53 sequences were amplified from pCV-GFP-N1 and pGBKT7–53 (Clontech) using primer pairs GFPF/POs20R and P53F/P53R (Table 1), respectively. The PCR products were then inserted into the pCMVTNT vector via the *Eco*RI/*Kpn*I or *Sal*RI/*Not*I sites, creating the control plasmids pCMV:GFP and pCMV:p53, respectively.

These PCR amplifications used LA *Taq* polymerase (TaKaRa Bio, Dalian, China), and the PCR amplification program as follows: preheating for one cycle of 3 min at 94 °C; 30 cycles of 30 s at 94 °C, 40 s at 58 °C, 1–3 min at 72 °C; and a final extension at 72 °C for 10 min. All clones derived from the PCR products were verified by sequencing, and the recombinant plasmids were confirmed by restriction analyses.

The *in vitro* pull-down assay was performed using a TNT Coupled Wheat Germ Extract System following the manufacturer’s recommendations (Promega). The plasmids for the generation of c-Myc-p53 fusion protein and GFP protein were used as negative controls in the experiments. *In vitro* translated c-Myc-RdRp^1–296^ (15 μL) and OsHSP20-GFP (15 μL) or NbHSP20-GFP (15 μL) were mixed and incubated at 4 °C overnight, then 20 μL of GFP-Trap M beads (ChromoTek, Martinsried, Germany) were added and incubated for another 6 h. The beads were washed four times with 0.5 ml washing buffer (0.05 M Tris-HCl, 0.15 M NaCl) and the bound complexes were eluted by boiling with 1 × protein loading buffer for 5 min. Samples were analyzed by western blotting as described elsewhere^38^. Briefly, Protein samples were separated by 12% SDS-PAGE gel electrophoresis and transferred by electroblotting to PVDF membrane (Millipore, Bedford, MA, USA). Transferred proteins were detected with anti-c-Myc or anti-GFP (monoclonal antibody; 1:2,000 dilution; Quanshijin, Beijing, China) primary antibodies and an anti-mouse horseradish peroxidase-conjugated secondary antibody (1:5,000 dilution; Kangweishiji, Beijing, China).

### Rice protoplast isolation and transfection

Rice protoplasts were isolated from 2-week-old seedlings as described^54^ with minor modifications. Briefly, young leaves and sheaths were chopped and dipped in enzyme solution (0.5 M mannitol, 1.5% cellulose RS (Yakult Honsa, Tokyo, Japan), 0.75% macerozyme R10 (Yakult Honsa), 1 mM CaCl_2_, and 0.1% BSA). This mixture was incubated on a shaking incubator (60 rpm) for 4 to 5 h at room temperature in the dark then filtered through Miracloth. Protoplasts were pelleted by centrifugation for 5 min at 200 *g* and resuspended in an equal volume of W5 solution (154 mM NaCl, 125 mM CaCl_2_, 5 mM KCl, and 1.5 mM MES, adjusted to pH 5.7). Protoplasts were centrifuged and re-suspended in MMG solution (0.4 M mannitol, 15 mM MgCl_2_, and 4.7 mM MES, adjusted to pH 5.7). Plasmid DNA (10 or 20 μg) was added to the protoplast solution and transfected with 40% polyethylene glycol (PEG) solution (40% PEG 4000, 0.4 M mannitol, and 100 mM Ca (NO_3_)_2_) for 20min at room temperature. W5 solution was added stepwise to dilute the PEG solution and discarded. Transfected protoplasts were incubated overnight at room temperature and then observed under confocal microscopy.

### Sub-cellular localization and BiFC assay

The binary expression vectors pCV-GFP-N1 and pCV-mCherry-N1, and the BiFC vectors pCV-nYFP-C and pCV-cYFP-C (for split YFP N-terminal/C-terminal fragment expression) were previously constructed in our laboratory^55^. For BiFC assays, the coding sequence of the N-terminal fragment (residues 1–296) of RSV RdRp was amplified using primer pair B1–1F/B1–1R (Table 1) and cloned into pCV-nYFP-C as a fusion with the N-terminal fragment of YFP via the *Bam*HI/*Sac*I sites, forming pCV-nYFP-RdRp^1–296^. The full-length coding sequences of OsHSP20 and NbHSP20 were amplified by PCR using primer pairs BOs20F/BOs20R and BNb20F/BNb20R (Table 1), respectively. PCR products were cloned into the *Bam*HI/*Sac*I or *Kpn*I/*Sac*I sites of pCV-cYFP-C as a fusion with the C-terminal fragment of YFP, resulting in pCV-cYFP-OsHSP20 and pCV-cYFP-NbHSP20, respectively.

For sub-cellular localization studies, the coding sequence of the N-terminal fragment (residues 1–296) of RSV RdRp was amplified using primer pair B1–1F/G1–1R (Table 1) and cloned into pCV-GFP-N1 and pCV-mCherry-N1 via the *Bam*HI/*Sal*I sites, forming pCV-RdRp^1–296^-GFP and pCV-RdRp^1–296^-mCherry, respectively. The full-length coding sequences of OsHSP20 and NbHSP20 were amplified by PCR with primer pairs BOs20F/GOs20R and BNb20F/GNb20R (Table 1), respectively. The products were subsequently digested with *Bam*HI/*Sal*I or *Kpn*I and ligated into the corresponding sites of pCV-GFP-N1, generating recombinant plasmids pCV-OsHSP20-GFP and pCV-NbHSP20-GFP, respectively.

For the BiFC assay in rice protoplasts, transient transfection of rice protoplast cultures with the combinations pCV-nYFP-RdRp^1–296^/pCV-cYFP-OsHSP20 and pCV-nYFP-RdRp^1–296^/pCV-cYFP-NbHSP20 were performed according to the protocol above, while the combinations pCV-nYFP-RdRp^1–296^/pCV-cYFP, pCV-nYFP/pCV-cYFP-OsHSP20 and pCV-nYFP/pCV-cYFP-NbHSP20 were used as the negative controls. For sub-cellular localization in rice protoplasts, the recombinant plasmids pCV-OsHSP20-GFP and pCV-NbHSP20-GFP were transfected into rice protoplasts, respectively. Fluorescence was detected in rice protoplasts 16–20 h after transfection.

The recombinant binary constructs above were introduced into *Agrobacterium tumefaciens* strain C58C1 by electroporation (Bio-Rad Gene Pulser, 0.2 cm cuvettes, 25 microF, >2.5 kV). Agroinfiltration was done as described^56^ with a few modifications. Briefly, cultures of C58C1 harbouring a relevant binary plasmid were grown in YEP medium containing rifampicin (50 μg ml^−1^) and kanamycin (100 μg ml^−1^) at 28 °C for 16 h. For the BiFC assay, C58C1 strains containing the BiFC plasmids were re-suspended and adjusted to an OD_600_ of 0.8:0.8 with infiltration medium (10 mM MES, pH 5.6, 10 mM MgCl_2_, 200 mM acetosyringone) before leaf infiltration. For sub-cellular localization, *Agrobacterium* cultures containing the pCV derivatives were re-suspended and diluted to an OD_600_ of 0.6 before leaf infiltration. The cell suspensions were incubated at room temperature for 2 to 4 h and then used to infiltrate 5- to 6-week-old *N. benthamiana* leaves. Expression of fluorescent proteins was examined at 48 h post agroinfiltration^57^.

### Confocal microscopy

Fluorescence analysis was performed using a Leica TCS SP5 confocal laser scanning microscope (Leica Microsystems, Heidelberg, Germany). GFP was excited at 488 nm and the emitted light captured between 500–550 nm, YFP was excited at 514 nm and the emitted light captured between 530–600 nm, and mCherry was excited at 561 nm and emission light captured between 570–630 nm. For analysis of co-localization assays, multi-tracking was used to prevent emission cross-talk between the channels. Images were captured digitally and handled using the Leica TCS software. Post-acquisition image processing was done with Adobe Photoshop version 7.0 software (Adobe Systems Inc., San Jose, CA, USA).

## Additional Information

**How to cite this article**: Li, J. *et al*. Interaction of HSP20 with a viral RdRp changes its sub-cellular localization and distribution pattern in plants. *Sci. Rep*. **5**, 14016; doi: 10.1038/srep14016 (2015).

## Supplementary Material

Supplementary Information

Supplementary Video S1

Supplementary Video S2

## Figures and Tables

**Table 1:** Primers used in plasmid construction.

Primers	Sequences (5′-3′)	Restriction sites (underlined)
R1-1F	GGAATTCCATATGACGACACCACCTCTCG	*Nde*I
R1-1R	CGCGGATCCTTACAGGACCCAGGTAGATCTG	*Bam*HI
R1-2F	GGAATTCCATATGAAACCAGGAGGAAGCAGA	*Nde*I
R1-2R	CCGGAATTCCTCGGTGGCAAGATCAGAT	*Eco*RI
R1-3F	CGCGGATCCCTAGCCATCTCATTGTCC	*Bam*HI
R1-3R	CGCGGATCCTGTCGTACAGGCAATCCA	*Bam*HI
R1-4F	GGAATTCCATATGACACCTTTGGGAGAGAAG	*Nde*I
R1-4R	CGCGGATCCGTTCTCCTTCATTAGCTG	*Bam*HI
R1-5F	CCGGAATTCACTCACCTAGGTGGTAGG	*Eco*RI
R1-5R	CGCGGATCCTCAGAAATCGAACTTATGGTC	*Bam*HI
Os20F	GGAATTCCATATGTCGCTGATCCGCCGCAG	*Nde*I
Os20R	CGCGGATCCCTAGCCGGTAACCTGGATG	*Bam*HI
Nb20F	GGAATTCCATATGTCTCTTATCCCAAGC	*Nde*I
Nb20R	CCGGAATTCTTAACCAGATATCTCAATGGC	*Eco*RI
B1-1F	CGCGGATCCATGACGACACCACCTCTCG	*Bam*HI
B1-1R	GCGAGCTCTTACAGGACCCAGGTAGATCTG	*Sac*I
BOs20F	CGCGGATCCATGTCGCTGATCCGCCGCAG	*Bam*HI
BOs20R	GCGAGCTCCTAGCCGGTAACCTGGATG	*Sac*I
BNb20F	CGGGGTACCATGTCTCTTATCCCAAGC	*Kpn*I
BNb20R	GCGAGCTCTTAACCAGATATCTCAATGGC	*Sac*I
G1-1R	ACGCGTCGACCAGGACCCAGGTAGATCTG	*Sal*I
GOs20R	ACGCGTCGACGCCGGTAACCTGGATGGAC	*Sal*I
GNb20R	CGGGGTACCACCAGATATCTCAATGGC	*Kpn*I
P1-1F	GGGGTACCATGGAGGAGCAGAAGCTGATC	*Kpn*I
P1-1R	ATAAGAATGCGGCCGCTTACAGGACCCAGGTAGATCTG	*Not*I
POs20F	CGGAATTCATGTCGCTGATCCGCCGCAG	*Eco*RI
POs20R	CGGGGTACCTTACTTGTACAGCTCGTCCATG	*Kpn*I
PNb20F	CGGAATTCATGTCTCTTATCCCAAGC	*Eco*RI
PNb20R	ATAAGAATGCGGCCGCTTACTTGTACAGCTCGTCCATG	*Not*I
GFPF	CGGAATTCATGGTGAGCAAGGGCGAGG	*Eco*RI
P53F	ACGCGTCGACATGGAGGAGCAGAAGCTGATC	*Sal*I
P53R	ATAAGAATGCGGCCGCTCAGTCTGAGTCAGGCCC	*Not*I
