# Supplementary material for: Interaction of HSP20 with a viral RdRp changes its sub-cellular localization and distribution pattern in plants
Source: Sci Rep. 2015 Sep 11;5:14016. doi: 10.1038/srep14016 (PMC4642574; doi:10.1038/srep14016)

**Interaction of HSP20 with a viral RdRp changes its sub-cellular localization and distribution pattern in plants**

**Jing Li** ^1^**, Cong-Ying Xiang** ^1, 2^**, Jian Yang** ^1^**, Jian-Ping Chen** ^1, *^**, Heng-Mu Zhang** ^1, *^

^1^ State Key Laboratory Breeding Base for Zhejiang Sustainable Pest and Disease Control, Key Laboratory of Biotechnology in Plant Protection of MOA and Zhejiang Province, Institute of Virology and Biotechnology, Zhejiang Academy of Agricultural Sciences, Hangzhou 310021, China

^2^ College of Chemistry and Life Science, Zhejiang Normal University, Jinhua 321004, China

* Corresponding authors, E-mail: [zhhengmu@tsinghua.org.cn](mailto:zhhengmu@tsinghua.org.cn); [jpchen2001@yahoo.com.cn](mailto:jpchen2001@yahoo.com.cn)

**Running Title: Interaction of HSP20 with a viral RdRp**

**Supplementary material**

**Figure S1** Microarray data analysis showing that transcripts of OsHSP20 were more frequent in plants infected by various viruses. (Data from [http://cdna01.dna.affrc.go.jp](http://cdna01.dna.affrc.go.jp/) and [http://rice.plantbiology.msu.edu](http://rice.plantbiology.msu.edu/))

**Figure S2** Sequence alignment (A) and similarity plot (B) of the OsHSP20 and its homologues from various species of plants.

**Figure S3** Investigation of RSV pc4-HSP20 interaction in YTH. Yeast colonies co-transformed with BD-pc4 and AD-OsHSP20, BD-pc4 and AD-NbHSP20, BD-OsHSP20 and AD-pc4 or BD-NbHSP20 and AD-pc4 showed no growth on the SD/-Ade/-His/-Leu/-Trp/X-α-Gal/AbA medium. Yeast co-transformed with BD-53 and AD-T, and BD-Lam and AD-T were used as the positive control and negative control, respectively.

**Video S1** The movement of OsHSP20-GFP granules in *N*. *benthamiana* epidermal cells.

**Video S2** The movement of NbHSP20-GFP granules in *N*. *benthamiana* epidermal cells.

Figure S1


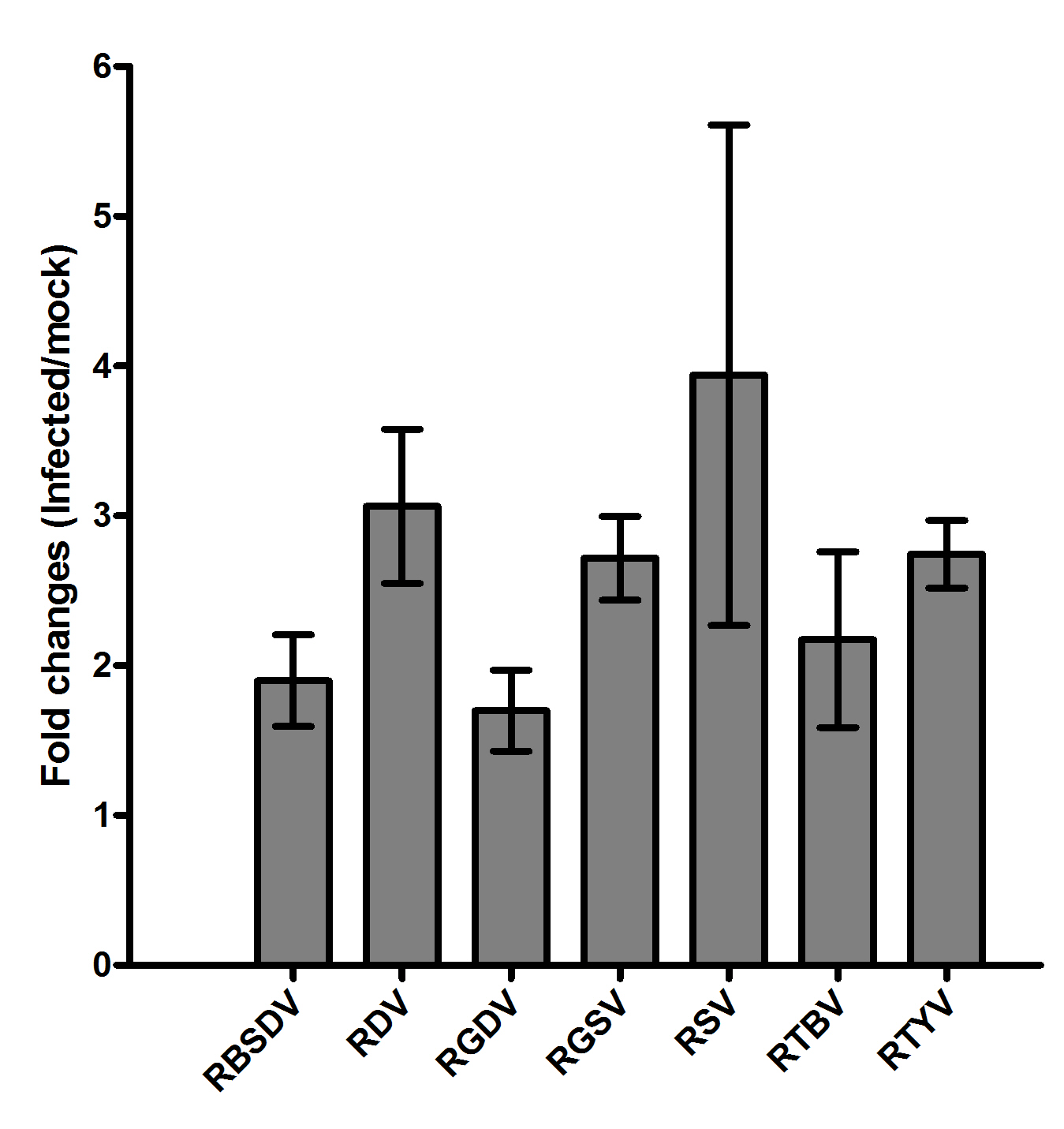


Figure S2


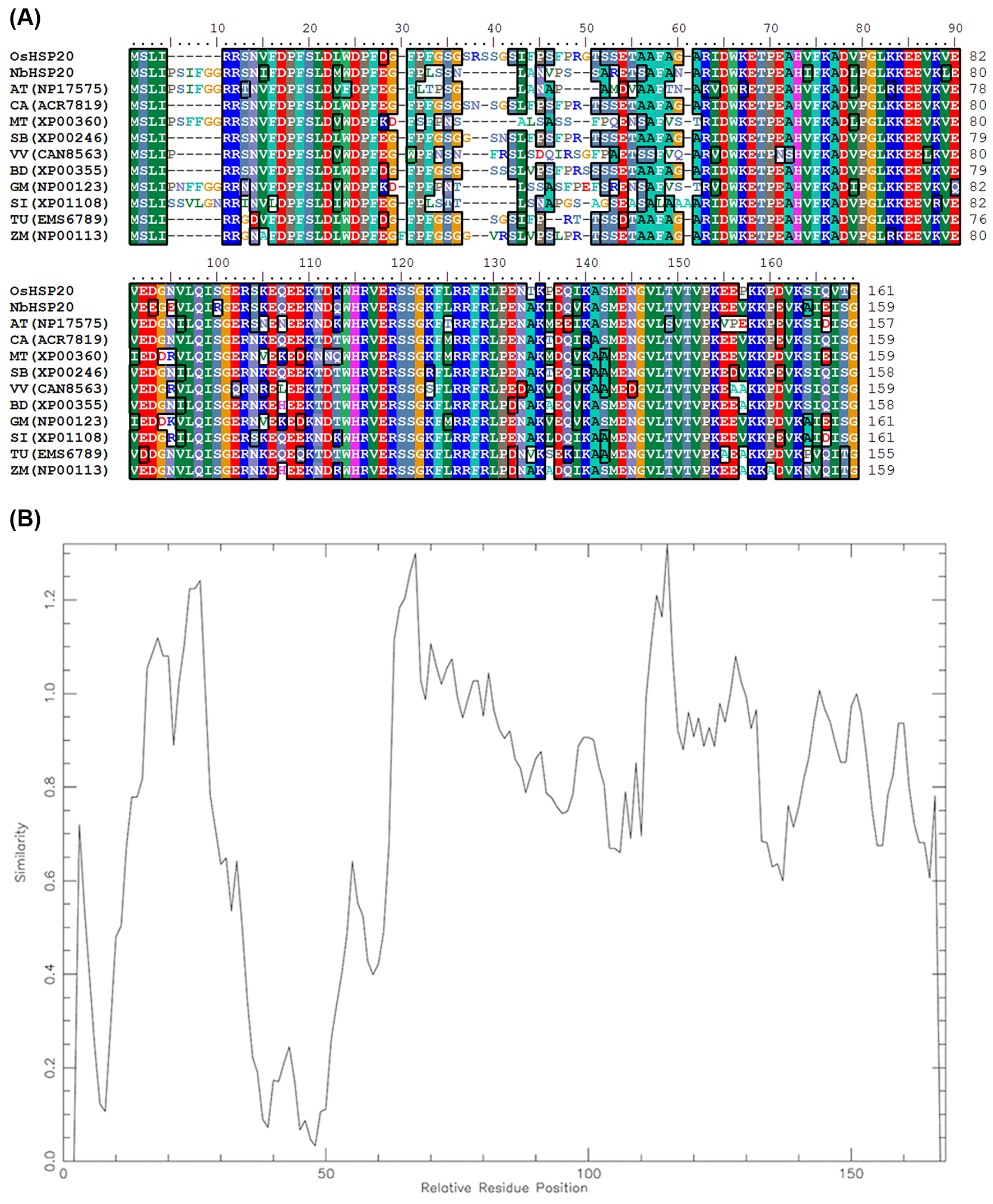


Figure S3


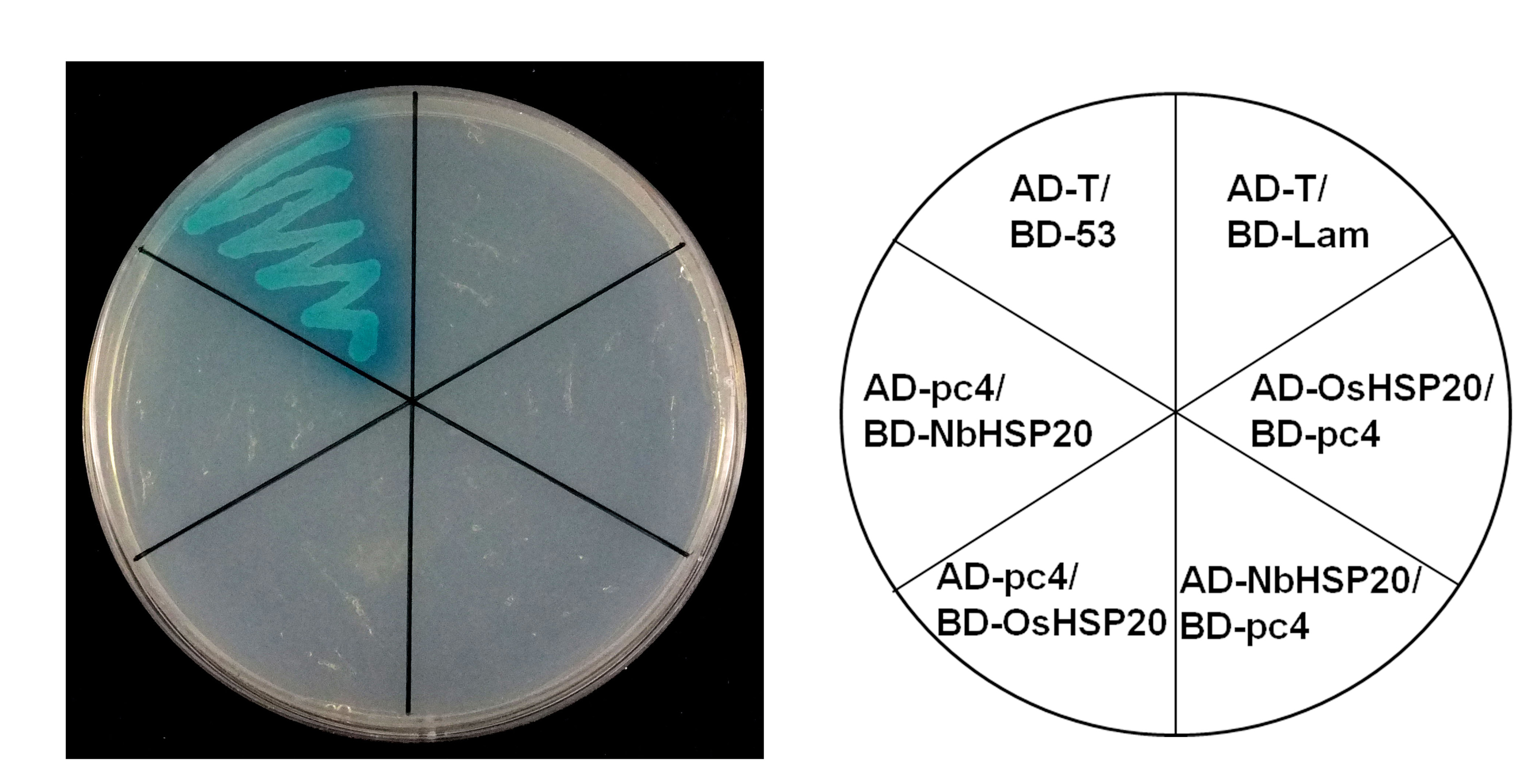

Supplement: Supplementary Information [file srep14016-s1.doc]
